# International Perspectives on COVID-19 Communication Ecologies: Public Health Agencies’ Online Communication in Italy, Sweden, and the United States

**DOI:** 10.1177/0002764221992832

**Published:** 2021-02-09

**Authors:** Serena Tagliacozzo, Frederike Albrecht, N. Emel Ganapati

**Affiliations:** 1 National Research Council of Italy, Institute for Research on Population and Social Policies, Rome, Italy; 2 Swedish Defence University, Stockholm, Sweden; 3 Florida International University, Miami, FL, USA

**Keywords:** COVID-19, communication ecologies, crisis communication, organizational communication

## Abstract

Communicating during a crisis can be challenging for public agencies as their communication ecology becomes increasingly complex while the need for fast and reliable public communication remains high. Using the lens of communication ecology, this study examines the online communication of national public health agencies during the COVID-19 pandemic in Italy, Sweden, and the United States. Based on content analysis of Twitter data (*n* = 856) and agency press releases (*n* = 95), this article investigates two main questions: (1) How, and to what extent, did national public health agencies coordinate their online communication with other agencies and organizations? (2) How was online communication from the agencies diversified in terms of targeting specific organizations and social groups? Our findings indicate that public health agencies relied heavily on internal scientific expertise and predominately coordinated their communication efforts with national government agencies. Furthermore, our analysis reveals that agencies in each country differed in how they diversify information; however, all agencies provided tailored information to at least some organizations and social groups. Across the three countries, information tailored for several vulnerable groups (e.g., pregnant women, people with disabilities, immigrants, and homeless populations) was largely absent, which may contribute to negative consequences for these groups.

Crises can place responding organizations under extreme pressure. To address crisis situations, responding organizations engage in communication networks that encompass a wide range of actors (Kapucu, 2006). Information in these networks flow in various directions: within and between organizations, as well as from organizations to the public and from the public to organizations (Quarantelli, 1997). However, these information streams are not isolated from each other. They constitute multilevel, interdependent communication ecosystems (Walter et al., 2018). The lens of communication ecology embraces this complexity, allowing researchers to explore the networks in communication ecosystems that individuals and organizations harness to reach their goals (Broad et al., 2013).

Utilizing a communication ecology approach in the context of the COVID-19 pandemic, we examine how, and to what extent, national-level scientific public health agencies deliver their communication in a coordinated and collaborative fashion with other organizations, including governmental, nongovernmental, scientific, or international organizations. We also explore whether scientific public health agency messages target different organizations and social groups. Studying these questions is important for several reasons. First, the crisis communication landscape is crowded by multiple organizations and actors that compete for public attention and legitimacy (Hall & Wolf, 2019; Holmes et al., 2009). Within this multivoice and information-rich environment, it is critical for responding organizations to collaborate with trusted partners for message delivery, in order to reduce information inconsistencies and cognitive stress in affected populations who receive conflicting messages (Hall & Wolf, 2019; Seeger, 2007). In this respect, without specific efforts to coordinate the communication, functional fragmentation of government agencies may pose the risk of multiplying the type of information provided to the public (Zeemering, 2020). Second, studies have demonstrated that message redundancy (i.e., receiving the same message from different sources) increases the possibility that the information is absorbed and acted on in crisis contexts (Stephens et al., 2013). Third, messages released in a coordinated and collaborative way avoid the creation of an information vacuum that may otherwise be filled by misinformation. Fourth, adequately tailored messages to different societal sectors or groups can promote an inclusive communication environment and address societal vulnerabilities that may place an undue burden (e.g., higher infection and death rates) on some social groups compared with others in the context of public health crises.

We compare communication ecologies of national-level public health agencies in Italy, Sweden, and the United States during the 3 months after the initial outbreak of COVID-19 in each country. This research contributes to the literature by focusing on public health agencies’ communication networks, which distinguishes it from previous studies that have generally examined the content of health risk communication (e.g., Crook et al., 2016; Rao et al., 2020). Furthermore, the comparative design of this study provides broader insights into the crisis communication field, where single case studies are more common, especially in the context of public health crises (e.g., D’Souza et al., 2020; Guidry et al., 2017; Rao et al., 2020; Roberts et al., 2017). Finally, the present study adds to the research literature by examining communication from public health agencies during emergencies, which has received less attention compared with other responding organizations (i.e., those in charge of emergency management).

Based on a descriptive content analysis of online COVID-19 communications in Italy, Sweden, and the United States through Twitter (*n* = 856) and press releases (*n* = 95), we find that health agencies relied heavily on their own scientific expertise and coordinated their communication efforts predominantly with external national government agencies during the initial months of the COVID-19 pandemic in each country. Targeted messages for several organizations and vulnerable social groups were remarkably absent from these agencies’ online communication, possibly yielding further negative consequences for these organizations and groups.

## Literature Review

Coordination and communication are key components of emergency management, and the research literature addressing these components is extensive. Focusing on interagency relationships (e.g., Comfort, 2007; Comfort et al., 2004; Kapucu, 2006), one stream of literature highlights the importance of effective communication and coordination with other relevant organizations to achieve common goals in the aftermath of a disaster. Despite this, organizations often encounter organizational, technical, and cultural barriers when they communicate with each other during disaster response (De Sisto & Handmer, 2020). Thus, forging partnerships before the disaster is critical to overcome some of these barriers (Kapucu, 2006).

Another stream of literature is dedicated to risk communication, often focusing on how communication is delivered to an external audience in terms of content, channels, and targets (e.g., Covello, 2003). The present research highlights the importance of disseminating information through multiple channels and tailoring content to the needs of a heterogeneous audience through targeted messaging (Kreuter et al., 1999). In this study, we adopt a communication ecology approach to analyze public health agency communication in terms of agencies’ engagement with other organizations and agencies’ target audience (i.e., to whom communication is directed). This approach highlights the interactions between the communication networks of organizations and individuals (Spialek & Houston, 2018) and, therefore, can partially bridge the aforementioned streams of literature.

Various guidelines recommend that parties involved in health risk communication should coordinate and jointly produce messages and specifically target vulnerable and at-risk populations (e.g., Arizona Department of Health Services, 2019; World Health Organization [WHO], 2020). This can help avoid fragmented or contradictory communication about health risks, as was the case in Italy (Missoni et al., 2020) and other countries during the COVID-19 pandemic. Zeemering (2020) noted that the collaboration with external actors as measured by, for example, Twitter’s mentions, could broaden the reach of city government agencies making the information about COVID-19 accessible beyond the council’s followers.

In public health crises, social media, such as Twitter and other online platforms, have become prominent channels for people to seek, receive, and share health messages. These media have been proven to be particularly useful in disseminating and monitoring health-related messages (Scanfeld et al., 2010). At the same time, these platforms have facilitated the propagation of misinformation (Kouzy et al., 2020) and multiplied the number of actors and nonexpert and nonindependent opinions in the health-related information arena. As a result, engaging with other organizations and groups has become increasingly critical for government and public health agencies.

Studies conducted on public health agencies’ communication in the context of prior public health crises have addressed how these agencies have used social media platforms (e.g., Guidry et al., 2017; Guidry et al., 2020; Ophir, 2019; Walton et al., 2012) or print media (e.g., Mebane et al., 2003), and how they responded to media inquiries (e.g., Robinson & Newstetter, 2003) or monitored communication from news outlets (e.g., Prue et al., 2003). Other research has focused on social media posts directed by the general public to public health agencies such as the U.S. Centers for Disease Control and Prevention (CDC; Crook et al., 2016), or has examined the public’s awareness of, trust in, and compliance with these agencies (e.g., Chon & Park, 2019; Kowitt et al., 2017). Additional research has examined the scientific integrity of health agencies and the loss of such integrity under a different political leadership (e.g., Goldman et al., 2020).

With respect to the measures of online engagement, previous studies have often used number of social media replies, mentions, and reuse of information (e.g., retweets; Liu & Xu, 2019; Wukich & Mergel, 2015). Zeemering (2020) comparatively interrogated coordinated communication about COVID-19 by local government agencies in three cities in the United States as measured by Twitter mentions. In the context of Twitter, previous analyses of engagement during disasters revealed that government organizations mainly rely on trusted institutional sources and amplify information provided by other government organizations (Sutton et al., 2015; Wukich et al., 2019; Wukich & Mergel, 2015). Liu and Xu (2019) found that emergency management agencies engage most with peer government agencies, citizen groups, and media organizations. Hence, organizational homophily is the main driver of collaboration in disaster situations, meaning that organizations are more inclined to collaborate within their sectoral boundaries (Sapat et al., 2019).

Despite extensive research on organizational communication and collaboration in the context of emergency management, few studies have analyzed the application of these recommendations. In particular, previous research has not clearly determined: (1) the extent to which coordination and collaboration among agencies is communicated to an external audience; and, (2) if communication is delivered in a coordinated and collaborative fashion. Hence, in this article, we examine two main research questions:

**Research Question 1:** To what extent have national-level scientific public health agencies delivered their communication in a coordinated and collaborative fashion with other organizations during the COVID-19 pandemic?
**Research Question 2:** Which organizations and social groups have scientific public health agency messages targeted during the pandemic?

## Methodology

### Cases

The present study investigates public health agencies’ online communication in three countries: Italy, Sweden, and the United States. While all three countries were affected by the COVID-19 pandemic, they adopted very different strategies to manage the crisis that ranged from keeping nearly all societal sectors open and operational to restrictions that approximated a general lockdown. According to the European Centre for Disease Prevention and Control (ECDC, 2020), as of September 20, 2020, there were a total of 332,440 identified cases in Italy as opposed to 94,987 in Sweden and 6,964,220 in the United States. These rates corresponded to 551, 928, and 2,104 identified cases per 100,000 population, respectively. The United States had the highest mortality rate per 100,000 population (61.71), followed by Italy (59.17) and Sweden (57.50; Johns Hopkins Coronavirus Resource Center, 2020).

The present study focuses on the three countries’ health agencies: the Italian National Institute of Health (*Istituto Superiore di Sanità*; ISS), the Public Health Agency of Sweden (*Folkhälsomyndigheten*), and the CDC in the United States. Given the differences in the political systems and political cultures across the countries, we expected to note several country-specific patterns. We were also interested in identifying some possible intercountry commonalities in the agencies’ communication, stemming from their common mission. Indeed, the three agencies are intentionally directed by scientific experts, implying that scientific assessments are the foundation for providing health information and recommendations to the general public and policy makers.

**Figure 1.**
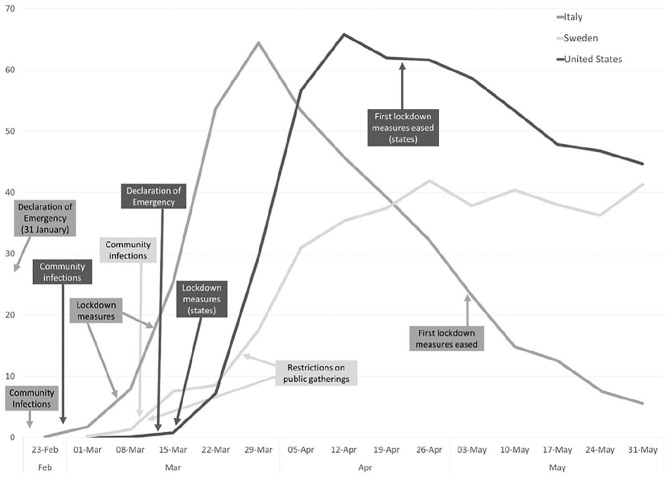
COVID-19 timelines and weekly case count per 100,000. *Note*. Source for case counts is ECDC (2020).

The study’s analysis period begins with the onset of community infections and lasts for a period of 3 months. As disease trends were unique for each country, the dates of study varied for each case, but represented the same stage of crisis management. For Italy, the analysis covered the period between February 21 and May 21, 2020. The Italian government initially adopted local quarantine measures and then extended the lockdown to the entire country. In May, these restrictions were slowly eased. For Sweden, we analyzed online communication from March 10 to June 10, 2020. Sweden kept most societal sectors open, but restricted public gatherings and introduced remote teaching in high schools and universities. In the United States, we examined the period 26 February 26 to May 26, 2020. Due to a decentralized government structure, COVID-19 policies (e.g., nonessential business closures, bans on large gatherings) were left to state and local governments. Figure 1 depicts the timeline of events and the extent of COVID-19 weekly cases during the selected time periods.

### Procedure

The study analyzed information published through Twitter and agency press releases. Twitter played a key role for the public and the government agencies to receive information in the past as well as during the current pandemic (e.g., Chew & Eysenbach, 2010; Rosenberg et al., 2020). Press releases were available on the agency websites, along with summarized information regarding disease trends and results of studies and projects. We examined a total of 856 Tweets exported from Twitter (Italy, *n* = 123; Sweden, *n* = 124; the United States, *n* = 609) utilizing the NCapture function of the qualitative data analysis software NVivo, combined with 95 press releases from agency websites (Italy, *n* = 22; Sweden, *n* = 59; the United States, *n* = 14).

Informed by a communication ecologies perspective, the content analysis of these sources aimed to determine the degree of engagement with other organizations and the extent to which communications were diversified by target population. To examine the agencies’ engagement with communication partners, we adapted the Martin et al. (2016) conceptualization of organizational engagement in disaster contexts that identified four levels of engagement: communication, cooperation, coordination, and collaboration. The nature of cooperation is short-term and weak, while coordination involves aligning actions to pursue a shared goal (e.g., through information sharing). The greatest level of engagement is collaboration, which refers to intentional, long-term relationships characterized by high levels of interdependency.

In this study, we focus on health agencies’ efforts to (1) communicate with other actors by *mentioning* them (e.g., mentions); (2) coordinate information with other parties through *information resharing* (e.g., retweet); and (3) cooperate with other actors through *collaborative efforts*. The category of *collaboration* includes sporadic cooperation and more established partnership that translates into information coproduction or collaborative projects. As for the target population, we divided it into two macrocategories: organizations (services/facilities) and social groups. Table 1 provides an overview of all variables and coding examples. We were guided by detailed coding instructions and coded all items abductively in three different languages. To increase intercoder reliability, we discussed unclear text and collaboratively agreed on the appropriate coding. The coding book for the study is available on request.

**Table 1.** Operationalization and Coding Examples.

Category	Description	Examples	Objective
Mentioning	The text mentions another actor but does not use or reuse information from this actor. Neither does the text indicate collaborative efforts between the actors.	“Every organization should follow the rules by the National Board of Health and Welfare.” (Folkhälsomyndigheten, May 7, 2020)	Mentioned Swedish National Board of Health and Welfare
Reusing or resharing information	The text directly reuses information from another actor, for example, through quoting or retweeting another actor. There is no indication of collaborative efforts.	Retweet of a tweet released by the Italian Ministry of Health: “Live Streaming of the press conference @istsupsan reporting the disease trends and the technical-scientific updates about #Covid19. (ISS, April 17, 2020)	Reused information from the Italian Ministry of Health
Collaborative efforts	Either information has been created collaboratively with another actor or it describes ongoing or planned cooperation with a partner.	“We know #COVID19 is causing anxiety for many of us. #BeKindToYourMind during these challenging times. CDC and @Google are partnering to share reliable, trusted information about ways to cope with stress.” (CDC, May 9, 2020)	Indicated collaboration with Google
Organizational targeting	The text targets an organization by directly addressing it or by providing information on activity carried out by this organization.	“CDC offers general considerations to help communities of faith decide how best to practice their beliefs while slowing the spread of #COVID19 and keeping their staff and congregations safe.” (CDC, May 23, 2020)	Was directed at faith organizations
Social group targeting	The text targets a social group by directly addressing it or by providing specific information to the group. (Exception: Where children are directly addressed, parents are regarded as the targeted group.)	“People older than 70 years should restrict social contacts until further notice.” (Folkhälsomyndigheten, March 16, 2020)	Was directed at elderly people

## Results

### Italy

#### The Italian National Institute of Health

The ISS is the leading scientific research agency providing counseling on public health matters to the Ministry of Health, the National Health Service, and the regional councils, as well as to Italian citizens and residents. Its work focuses on preventing diseases, promoting healthy behaviors, treating chronic illness, and controlling the quality and safety of health technologies. The ISS has a website and social media accounts on YouTube and Twitter. It also provides information through a knowledge management platform (*ISS Salute*) and an online journal (*Epicentro*).

#### Online COVID-19 Communication Ecologies

##### Engagement with organizations

**Figure 2.**
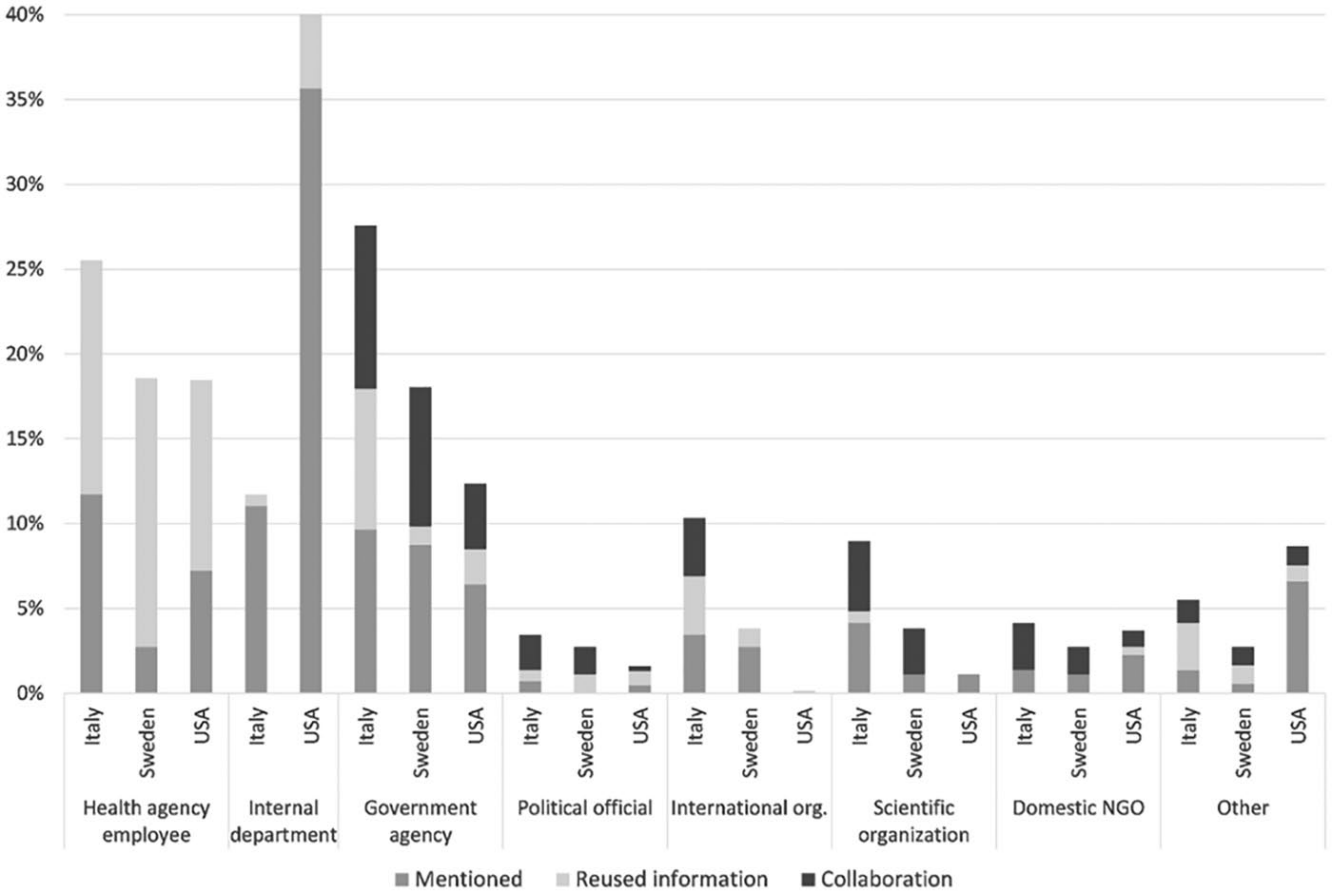
Engagement with organizations.

Results of the analysis of the engagement levels (mentions, reuses, and collaborations) revealed that the ISS engaged mainly with external government organizations (27.6%) and internal employees (25.5%), whereas the least engaged actors were political officials and domestic nongovernmental organizations (NGOs; see Figure 2). Closer examination of different engagement levels revealed that collaborations with external organizations (thus, excluding internal employees and departments) were mainly established with government organizations (9.7%) and scientific institutions (4.1%). The Italian Ministry of Health was the government organization with which the ISS engaged and collaborated the most. As for the reuse of information, in 13.8% of the cases the agency reused information (e.g., quoting a sentence) produced by an ISS employee, and in 8.3% of cases, reused communication from an external government agency (e.g., through retweets). Most of the information reused was originally produced by the Ministry of Health. As for the mentions, in most of the cases the agency mentioned an internal employee (11.7%) or a department (11%), followed by the 9.7% of the cases in which a national government organization was mentioned.

##### Targeted organizations and groups

**Figure 3.**
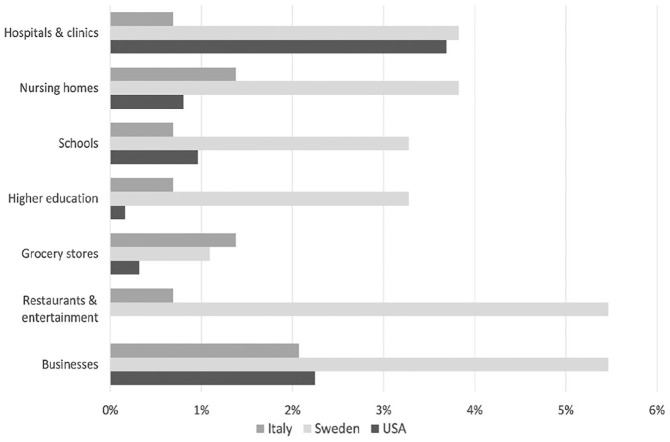
Organizations targeted by online communication (selected).

**Figure 4.**
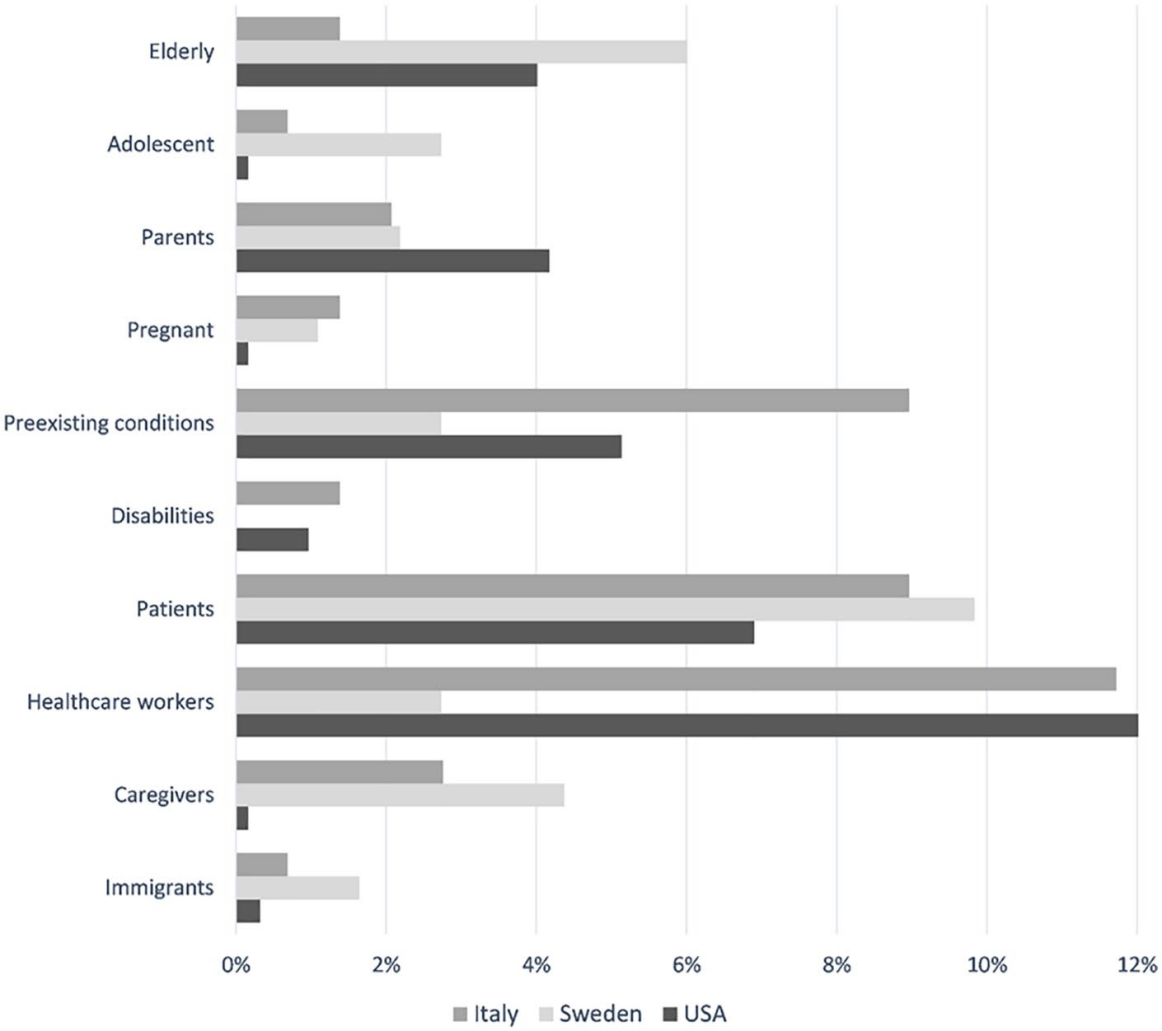
Social groups targeted by online communication (selected).

As far as the targets of the communication were concerned, facilities and public services were addressed sparingly (see Figure 3). The most represented categories were businesses (2.1%) groceries (1.4%), and nursing homes (1.4%). Only one case was reported for hospitals, schools, higher education facilities, and restaurants/entertainment facilities. No information was directed to correctional and mass transit facilities or to faith-based organizations or child care and emergency services. In all, messages were formulated for a general audience (64.1%) containing information (e.g., information about disease trends) that could be relevant for anyone. The social groups most frequently targeted consisted of health care workers (11.7%), people with medical preconditions (9%), and COVID-infected patients (9%). The least represented categories encompassed caregivers (2.8%), parents (2.1%), adolescents, pregnant women, people with disabilities, migrants, and elderly people (all below 2%). No messages were aimed at young adults or homeless people (see Figure 4).

### Sweden

#### The Public Health Agency of Sweden

The Public Health Agency of Sweden (Folkhälsomyndigheten) is a government agency with scientific expertise on diseases and public health concerns. The agency provides information to the government, which is used as a decision basis for policy makers. The agency is also responsible for implementing policies decided by the government, but it acts independently within given directives. Furthermore, the agency communicates with other agencies on local, regional, and national levels, and communicates with the general public. Its vision is to achieve “a public health that strengthens the positive development of society” (Folkhälsomyndigheten, 2016, p. 4). During the COVID-19 pandemic and previous health crises, scientific experts in the agency played a dominant role in disseminating information to the public and recommendations to the government (Giritli Nygren & Olofsson, 2020). The agency provides information on social media (Facebook, Twitter, and YouTube) and through the agency website (in the form of a webpage section and press releases). The agency also streamed daily collaborative press conferences live on YouTube during the initial 3 months of the pandemic.

#### Online COVID-19 Communication Ecologies

##### Engagement with organizations

The online communication from the Public Health Agency of Sweden mostly consisted of information from their own employees, that is, social media mentions and reuse of information they provided in interviews (18.6%). Apart from internal sources, the agency engaged and collaborated frequently with other government agencies (18%). Engagement with other organizations was low (see Figure 2). In the agency’s online communication, the least visible engagement included communication with domestic NGOs (2.7%), scientific authorities at the domestic (3.8%) and international levels (3.8%), and political officials (2.7%).

##### Targeted organizations and groups

The agency targeted multiple organizations directly in online communication, such as restaurants, schools, institutions of higher education, as well as health care facilities and nursing homes (see Figure 3). However, the agency rarely addressed other essential organizations, such as grocery stores and mass transit companies (1% and below). Most (80.3%) messages included information directed at the general public. Tailored information was also directed specifically at several social groups, but not all social groups were considered equally. Most frequently targeted were those expressing COVID-19 symptoms, elderly people, and their caregivers (see Figure 4). Conversely, pregnant women, immigrants, and people with disabilities rarely received tailored information in tweets and press releases (1.1%, 1.6%, and 0%, respectively). Furthermore, of the three messages directed at immigrants, only one was available in a language other than Swedish.

### The United States

#### Centers for Disease Control and Prevention

The CDC is a public health agency housed under the Department of Health and Human Services (DHHS). The agency’s mission highlights its national role in “developing and applying disease prevention and control, environmental health, and health promotion and health education activities designed to improve the health of the people of the U.S.” (CDC, 2020). The CDC also provides assistance on disease prevention and control to agencies across different sectors as well as international agencies and other nations. The CDC is active on the social media sites of Facebook, Twitter, and YouTube, and it maintains complementary Spanish versions of its web content for Spanish speaking populations.

#### Online COVID-19 Communication Ecologies

##### Engagement with organizations

The CDC’s online COVID-19 communication revealed relatively limited collaboration between the agency and other actors. Only a small amount of all communication expressed collaboration. The collaboration was mainly with officials within the DHHS (7.2%) and external government agencies (3.9%), followed by the *other* category (e.g., Google, Fox News Radio; 1.1%). Within the reuse category, the top subcategory was DHHS officials (11.2%) and the external government agencies (2.1%). A higher share of CDC communication mentioned other agencies compared with the share that indicated collaboration or reused information. DHHS officials (7.2%) and external government agencies (6.4%) were the most mentioned among all subcategories. International organizations were missing from the CDC’s online communication, except for one tweet. Other scientific authorities received little attention in the agency’s communication. Only 1.1% of CDC communication mentioned such authorities, and none was mentioned as a collaborative partner. The CDC directed relatively more attention to domestic NGOs (4.14%) and political officials (1.6%) by mentioning or indicating collaboration with them compared with the attention it directed toward international agencies and scientific authorities.

##### Targeted organizations and groups

The CDC directed a small share of information to organizations. In the United States, most of this communication targeted the hospitals and clinics subcategory (3.7%), followed by the businesses subcategory (2.2%) and the *other* subcategory (1.1%). The *other* subcategory includes diverse organizations ranging from critical infrastructure industries and organizations that provide critical functions to homeless shelters and camps. In the CDC’s online communication, mass transit agencies received the least attention—no communication targeted or included reference to these agencies. Apart from a single targeted communication for each, higher education organizations and restaurants were ignored in CDC communication. There was also limited engagement with facilities that house populations at higher risk for COVID-19, such as correctional facilities (1%), nursing homes (0.8%), and retirement communities (0.3%).

As for the social groups, among all CDC communication, 63.4% targeted the general public and 21% was in the *other* subcategory, which included groups ranging from family members of COVID-19 patients and deaf persons to crew members of cruise lines and poll workers. In terms of specific social groups, the agency’s top targeted audience included health care workers (12%), COVID-19 patients (6.9%), people with preexisting health conditions (5.1%), parents (4.2%), and elderly populations (4%). Disabled persons and young adults were targeted relatively little: 1% and 0.6%, respectively. Caregivers, pregnant women, and adolescents received even fewer targeted messages (0.2% or less).

### Comparison of Cases

#### Engagement With Organizations

Overall, we found a higher level of engagement with other organizations in Italy than in Sweden or the United States. Figure 2 depicts engagement with communication partners. In all cases, internal sources were mentioned frequently, and information from internal sources was reused often (Italy: 25.5%, Sweden: 18.6%, the United States: 18.5%). In addition, the CDC engaged with internal partners within the same umbrella organization—that is, DHHS (8.8%). Comparable internal organizations do not exist in Italy or Sweden.

Public health agencies engaged with external government agencies as well, although the levels of engagement and collaboration varied between the countries. The ISS in Italy mentioned and reused information from other government agencies and collaborated with them more frequently, but government agencies were essential communication partners in all studied countries (Italy: 27.6%, Sweden: 18%, the United States: 12.4% [excluding organizations within DHHS: 8.8%]). Engagement with political officials was similarly low in all three countries (Italy: 3.5%, Sweden: 2.7%, the United States: 1.6%). In Italy, we found frequent engagement with international organizations such as the WHO and ECDC and domestic scientific authorities, whereas this was less common in Sweden and scarce in the United States (Italy: 19.3%, Sweden: 7.7%, the United States: 1.3%). Finally, in all countries, domestic NGOs were rarely engaged (Italy: 4.1%, Sweden: 2.7%, the United States: 3.7%).

In sum, despite expected differences between the countries, we identified several commonalities in the communication delivered by national-level public health agencies. First, these agencies mostly relied on internal actors to coordinate communication, either through the health agency’s own staff or internal organizations (e.g., CDC Travel). Second, the health agencies interacted frequently with external government agencies, but kept interaction with political officials to a minimum. Third, the agencies rarely engaged with domestic NGOs in communications relating to COVID-19.

#### Targeted Organizations and Groups

Targeted information to specific or social groups varied between the countries (see Figure 3). For instance, the Public Health Agency of Sweden targeted more different types of organizations (e.g., education institutions, restaurants, and health care and care facilities), whereas its U.S. counterpart focused on health care facilities and businesses. In Italy, the ISS provided overall less targeted information to different organizations. Interestingly, in all countries, organizations representing essential services in the context of the pandemic (e.g., child care facilities, mass transit organizations, and grocery stores) were rarely or never targeted in health agency messages (1.4% and below).

Social groups were targeted to a similar extent in all three countries, but there were variations between countries. Patients were targeted frequently by all three agencies, but Italy and the United States provided more tailored information to health care workers and people with preexisting health conditions that are at higher risk.^1^ In contrast, all three countries showed remarkable similarities with regard to social groups neglected by agency messages. For example, information provided on Twitter and in press releases was rarely or never directed at pregnant women, people with disabilities, immigrants, and homeless populations (see Figure 4).

## Discussion

Our analysis revealed several differences and some commonalities in the communication ecologies of health agencies across the three countries during the initial 3 months of the pandemic. Each country is unique in terms of its emergency management, public health and government systems, and social and cultural contexts, so interpreting this study’s findings was challenging. However, in this discussion, we propose possible explanations that will need to be examined in future studies.

To begin, our study illustrates that public health agencies in Italy, Sweden, and the United States heavily relied on their own scientific expertise to disseminate COVID-19 information to the public. In the case of the CDC, our finding on the extent of the agency’s reliance on its own expertise aligns with Roberts et al. (2017), who found that the CDC often provided hyperlinks directed to its own website in the context of the Ebola epidemic. Several factors might explain this, including an inward-oriented organizational culture as opposed to one that is more outward looking (Grönroos, 2019; Waterhouse & Lewis, 2004). Another factor might be that public health agencies wanted to amplify their messaging through science-based primary data in a highly uncertain context such as the COVID-19 pandemic.

In addition to engagement via internal employees, the high level of engagement with other national government organizations in all three countries indicates that these entities play an important role within the health agencies’ communication ecosystem. Similar patterns of online engagement behaviors were identified by previous literature (Liu & Xu, 2019; Sutton et al., 2015; Wukich & Mergel, 2015; Zeemering, 2020) in which government and emergency agencies proved to engage predominantly with other government organizations. This tendency of health agencies to partner mainly with internal departments or external trusted government partners in their communicative efforts may come at the expense of other organizations. Indeed, domestic NGOs were rarely included in the agencies’ communication, implying that they were arguably not seen as relevant sources of information or collaboration partners. Johnson Avery and Kim (2009) noted a general tendency of health agencies such as the WHO and the CDC to make reference to governmental and public partners and exclude private sector organizations in their press releases. On the contrary, previous research and empirical evidence have suggested that NGOs have played a key role providing critical services to those in need during the COVID-19 pandemic, especially to socially vulnerable groups (Akingbola, 2020). Thus, it seems reasonable to conclude that engaging more frequently with NGOs may increase the health agencies’ understanding of different social groups and help NGOs cater to vulnerable groups they serve.

Compared with the other two countries, Italy’s higher engagement with international organizations such as the WHO and ECDC, as well as domestic scientific authorities, can be explained by the need for assistance and initial guidance to manage an unprecedented crisis. Indeed, Italy was the first European country to be affected by the pandemic (Vagnoni, 2020), so the country was unable to pattern its response based on the experiences of other countries. Although the COVID-19 outbreak originated in China, the country’s authoritarian government disclosed limited information on the outbreak and how to mitigate it initially (Zhang et al., 2020). Hence, Italy may have needed to draw expertise from international organizations, such as the WHO, which had extensive experience in responding to public health crises, such as the H1N1, severe acute respiratory syndrome (SARS), and the Ebola outbreaks. Conversely, Sweden and the United States managed the pandemic in different ways: Sweden pursued a soft approach built on individual responsibility and voluntary recommendations, which was criticized by international actors (Giritli Nygren & Olofsson, 2020). In the United States, crisis management was left to states and local decision makers. Both management styles may have led to decreased engagement with international organizations.

Regarding the information targets, some notable differences must be highlighted. Unlike the ISS and the CDC, the Swedish agency had a higher percentage of messages targeting different types of organizations. This might be explained by Sweden’s decision to keep borders, schools (under the age of 16 years), and many businesses, including restaurants and bars, open and operational (instead of pursuing lockdown measures that were applied in Italy and parts of the United States) with the resulting need to maintain open communication channels with businesses and services. Some key organizations or services that had to remain fully operational during the peak of the pandemic, such as mass transit facilities, correctional services and grocery stores, were rarely targeted by communications from the health agencies in any of the countries. It is possible that the public health agencies assumed that individual responsibility weighs most in these contexts, especially in Sweden (Giritli Nygren & Olofsson, 2020). The agencies might have considered that their agencies’ charge did not go beyond communicating with the general public that use these organizations’ facilities or services. Furthermore, public health agencies might have perceived these organizations as falling outside their area of responsibility or they might have established other communication channels with these organizations. Limited communication with these organizations might also be explained by negative social construction of population groups using these facilities (e.g., how low-income individuals and prisoners are portrayed in negative terms; Schneider & Ingram, 1993).

The analysis also indicates clear differences in the degree to which social groups were targeted by the three agencies. Some differences (e.g., targeting of health care workers) may be explained by how the public health system in each country is structured. For example, in Sweden, the health care sector is managed on a regional level in collaboration with national government agencies other than the Public Health Agency of Sweden. Thus, guidelines for health care workers do not fall under the jurisdiction of the health agency. When targeting elderly people, considerations regarding the *digital divide* (i.e., technology use is less common in older vs. younger populations) may have affected agencies’ online communication in Italy and the United States (Colombo et al., 2018), the elderly social group was targeted at low rates in these communications. Conversely, in Sweden, 96% of people between the ages 65 and 74 years, and 77% of people between the ages 75 and 84 years, use the internet (Statistiska centralbyrån, 2020). In addition, half of all people 70 years and older used the internet to retrieve information on the COVID-19 pandemic regularly, possibly resulting in a greater need to communicate with them through web-based tools (Gustavsson & Beckman, 2020). Another explanation may be found in the Swedish strategy, which strongly focused on the protection of elderly people as a core vulnerable group, accompanied with repeated and specific recommendations for elderly people to self-isolate.

Despite these significant variations in the most targeted social groups, we found evidence for several commonalities across countries. Unsurprisingly, people with COVID-19 symptoms were targeted frequently by all agencies. More important, however, we found that the agencies did not often tailor information for groups such as pregnant women, people with disabilities, immigrants, and homeless populations. These groups are generally regarded as the particularly vulnerable in cases of a disaster or crisis (Tierney, 2014), and studies have repeatedly highlighted the importance of their preparedness for public health emergencies (Andrulis et al., 2007). This result echoes the findings by Sutton et al. (2020), who analyzed Twitter communications by governmental public health agencies in the United States during the first 2 months of the pandemic noting the absence of hashtags specifically directed to at-risk groups. Tailored messages, as opposed to broad messages targeting the general public, are needed to reach different social groups, specifically in the context of health communication (Kreuter et al., 1999). Public health agency communication strategies must provide vulnerable groups with relevant, adequate, and specific information; if vulnerable groups are excluded or not targeted, their capacity to prepare for and respond to crises may be negatively affected, ultimately exacerbating existing social inequalities. The studied agencies may have provided tailored information to vulnerable groups on other platforms; however, individuals from these groups would need to commit additional time and effort (e.g., active search in the webpages) to access such information.

This study is restricted to data from Twitter and agency press releases. Future research should include additional information sources (e.g., Facebook, agency websites) to provide a more comprehensive understanding of public health agencies’ online communication. Future studies should also compare the agencies’ COVID-19 communication to their pre-COVID-19 communication patterns to highlight significant shifts and to understand similarities and differences in communication trends in other public health emergencies (e.g., H1N1 or Ebola). Furthermore, our study examined communication efforts from the perspective of health agencies. Additional studies could examine how these agencies’ communications are perceived and acted on by their audience.

## Conclusion

This article provides a comparative analysis of online communication ecologies of public health agencies in Italy, Sweden, and the United States. Findings indicate that, although the communication ecosystems varied across the countries, there were several noteworthy commonalities. In particular, we found similar patterns in the type of organizations that public health agencies engaged and collaborated with and the type of organizations and groups that were mostly ignored by these communication ecosystems. Overall, the study illustrates that some features of these communication networks persist across geographical and organizational boundaries. These “closed” communication ecosystems may exclude other relevant partners and groups from communication activities and ultimately undermine the efficacy of the communication efforts.

The identification of organizations and groups that are neglected across cases is a remarkable result because it illustrates broader trends. First, while agencies heavily rely on internal sources and external government agencies, other organizations (e.g., domestic NGOs) were underrepresented in all three cases. Given the essential role many NGOs fulfill during crises, interaction with these organizations may improve agency communication and create more inclusive communication ecosystems. Second, social groups that were rarely or never targeted in communication efforts belong to groups that have been previously identified as particularly vulnerable to disasters. It is crucial to provide them with tailored and adequate information to increase their access to relevant information and improve their coping capacities. Lack of tailored information for vulnerable groups indicates a need to adapt communication strategies and implement policies that focus on including vulnerable social groups more adequately in communication efforts.

## Notes

1. Information for health care workers is generally disseminated by other government agencies in Sweden; the Public Health Agency of Sweden is not responsible for conveying this information.
